# Intestinal Dysbiosis Is Associated with Altered Short-Chain Fatty Acids and Serum-Free Fatty Acids in Systemic Lupus Erythematosus

**DOI:** 10.3389/fimmu.2017.00023

**Published:** 2017-01-23

**Authors:** Javier Rodríguez-Carrio, Patricia López, Borja Sánchez, Sonia González, Miguel Gueimonde, Abelardo Margolles, Clara G. de los Reyes-Gavilán, Ana Suárez

**Affiliations:** ^1^Department of Microbiology and Biochemistry of Dairy Products, Instituto de Productos Lácteos de Asturias (IPLA-CSIC), Villaviciosa, Asturias, Spain; ^2^Area of Immunology, Department of Functional Biology, University of Oviedo, Oviedo, Asturias, Spain; ^3^Area of Physiology, Department of Functional Biology, University of Oviedo, Oviedo, Asturias, Spain

**Keywords:** free fatty acids, systemic lupus erythematosus, dysbiosis, microbiota, short-chain fatty acids

## Abstract

Metabolic impairments are a frequent hallmark of systemic lupus erythematosus (SLE). Increased serum levels of free fatty acids (FFA) are commonly found in these patients, although the underlying causes remain elusive. Recently, it has been suggested that factors other than inflammation or clinical features may be involved. The gut microbiota is known to influence the host metabolism, the production of short-chain fatty acids (SCFA) playing a potential role. Taking into account that lupus patients exhibit an intestinal dysbiosis, we wondered whether altered FFA levels may be associated with the intestinal microbial composition in lupus patients. To this aim, total and specific serum FFA levels, fecal SCFA levels, and gut microbiota composition were determined in 21 SLE patients and 25 healthy individuals. The *Firmicutes* to *Bacteroidetes* (F/B) ratio was strongly associated with serum FFA levels in healthy controls (HC), even after controlling for confounders. However, this association was not found in lupus patients, where a decreased F/B ratio and increased FFA serum levels were noted. An altered production of SCFA was related to the intestinal dysbiosis in lupus, while SCFA levels paralleled those of serum FFA in HC. Although a different serum FFA profile was not found in SLE, specific FFA showed distinct patterns on a principal component analysis. Immunomodulatory omega-3 FFA were positively correlated to the F/B ratio in HC, but not in SLE. Furthermore, divergent associations were observed for pro- and anti-inflammatory FFA with endothelial activation biomarkers in lupus patients. Overall, these findings support a link between the gut microbial ecology and the host metabolism in the pathological framework of SLE. A potential link between intestinal dysbiosis and surrogate markers of endothelial activation in lupus patients is supported, FFA species having a pivotal role.

## Introduction

Epidemiological studies have consistently shown an increase in the prevalence and severity of a number of metabolic disorders in patients with systemic lupus erythematosus (SLE) compared to the general population (1–3). Among them, metabolic syndrome, disturbed glucose metabolism, or altered lipid metabolism are the most relevant. These disorders are related to an increased risk of cardiovascular disease (CVD) development, the most important cause of mortality in SLE (4, 5), thus highlighting the clinical relevance of the metabolic alterations in SLE.

Immune dysregulation and chronic inflammation are known to promote endothelial dysfunction in SLE (6, 7). Increased levels of pro-inflammatory cytokines [such as tumor necrosis factor alpha (TNFα), interleukin-8 (IL-8), and monocyte chemoattractant protein-1 (MCP-1)], adipokines, and autoantibodies are associated with the progression of endothelial dysfunction toward atherosclerosis development (6). At the local level, these mediators can impair the balance between endothelial repair and damage, whereas a number of systemic effects can be also triggered, including a shift to a pro-oxidant status (8) and altered lipid metabolism. In this scenario, although the underlying mechanisms are not totally understood, the relationship between systemic inflammation, metabolic disorders, and CVD may be explained, at least in part, by the free fatty acids (FFA) (9). FFA are fatty acid molecules released from adipocytes and several cell types upon lipolysis (10). Increased FFA levels in serum have been described in several metabolic disorders. Moreover, elevated serum FFA have also been found in immune-mediated diseases, such as SLE or rheumatoid arthritis, although striking differences were noted between both conditions (11). Rather than inflammatory or clinical parameters, the body mass index (BMI) was found to be the main predictor of FFA serum levels in lupus (11). However, these clinical studies did not allow the elucidation of the exact mediators and mechanisms involved.

Obesity is the result of an imbalance between energy intake and expenditure, which results in an excess of fat accumulation. However, several epidemiological studies have identified people with low BMI exhibiting markers of metabolic dysfunction (12, 13). Similarly, healthy metabolic profiles are found in a subset of obese subjects (14, 15), hence suggesting that metabolic dysfunction (that is, impaired fatty acid mobilization) rather than adiposity should be considered as the underlying cause. Therefore, it is feasible that factors related to energy intake and expenditure may underlie altered FFA levels and thus, metabolic disorders.

A mounting body of evidence shows that the gut microbiota can influence the host metabolism as well as the energy harvest and storage (16–18). Actually, the gut microbiota is seen by some authors as a separate endocrine organ involved, through a molecular cross talk with the host, in the maintenance of energy homeostasis and fat deposition (19). Currently, extensive research efforts have been focused on deciphering the basis of the cross talk between the microbiota and the host metabolism in the development and progression of host diseases and have revealed the relevance of the intestinal microbiota–host metabolism axis mediated by different bacterial and host metabolites (20, 21). Thus, it may be speculated that changes in the intestinal microbial ecology could disrupt this homeostatic cross talk and precipitate the development of pathological outcomes in the host.

Recently, we have reported that SLE patients exhibit an altered intestinal composition compared to healthy subjects, mainly characterized by a decreased abundance of members of the *Firmicutes* phylum and an overrepresentation of those of *Bacteroidetes* (22). However, the clinical impact of this SLE-associated intestinal dysbiosis remains to be elucidated. Taking into account the former assumptions, we hypothesized that altered gut microbiota composition in SLE may underlie increased FFA serum levels. Accordingly, the main aims of the present report were (i) to analyze the potential association between the microbiota composition and FFA serum levels, (ii) to elucidate whether microbial metabolites can have a role in this interaction, (iii) to evaluate whether a different profile of FFA can be found in lupus patients, and (iv) to study the associations of these parameters with clinically relevant serum biomarkers.

## Materials and Methods

### Ethical Approval

Ethical approval for this study was obtained from the Institutional Review Board (Comité de Ética de Investigación Clínica del Principado de Asturias) in compliance with the Declaration of Helsinki. All participants were informed and gave a signed informed consent prior their inclusion in the study.

### Patients and Controls

Our study involved 21 SLE patients, all fulfilling classification criteria for SLE. According to the 1982 revised criteria from the American College of Rheumatology, a definitive SLE diagnosis can be established when a patient exhibit at least 4 out of the 11 SLE criteria (malar rash, discoid lesions, photosensitivity, oral ulcers, arthritis, serositis, renal disorders, neurological disorder, cytopenia, raised anti-DNA titers, and positivity to antinuclear antibodies) (23). A complete clinical examination, including Systemic Lupus Erythematosus Disease Activity Index (SLEDAI) calculation and anti-dsDNA autoantibodies assessment, was performed at the time of sampling. All patients were in remission (SLEDAI <8) at the sampling time. Information on clinical features along the disease course as well as the therapies received during the last 6 months was obtained from their clinical records. A group of 25 age- [median 43.50 (range 23.00–63.00) years] and gender-matched (23 females) healthy individuals recruited from the general population was included as the healthy controls (HC). Patients and controls did not differ in age (*p* = 0.293) and gender distribution (*p* = 0.495). Exclusion criteria were the history of recent infections, diagnosis of metabolic diseases, or the use of antibiotics, glucocorticoids, or monoclonal antibodies in the previous 6 months.

Upon acceptance of the individuals to participate in the study, a strict overnight fast (more than 8 h) blood sample was obtained in tubes without anticoagulant. Serum was collected, divided into aliquots, and samples were stored at −80°C until experimental procedures were performed. Additionally, basic serum blood lipid analyses were carried out on fresh samples at the time of sampling, by standardized procedures.

### Quantification of Total FFA Serum Levels

Total FFA serum levels were analyzed by a colorimetric enzymatic assay using a commercial kit (NEFA kit half-microtest, Roche Life Sciences, Penzberg, Germany) following the instructions from the manufacturer. Final absorbance was measured at 546 nm, and the detection limit was 0.2 mM.

### Assessment of Serum FFA Profiles

Individual FFA were analyzed in serum samples following a methyl-*tert*-butylether-based extraction protocol (MTBE) as previously described (24), with minor modifications. Briefly, serum samples (100 µl) were spiked with 5 µl of internal standard (600 ppm heptadecanoic acid). Proteins were precipitated by the addition of 200 µl methanol chromasolv grade (Sigma Aldrich, MO, USA). Organic phases were obtained by the addition of 1,200 µl MTBE chromasolv grade (Sigma) followed by an incubation in an ultrasound water bath at 15°C for 30 min. Finally, organic phases were isolated by centrifugation at 5,000 rpm (7 min, 15°C) after the addition of 200 µl milliQ water. The extraction protocol was repeated once with 100 µl MetOH, 500 µl MTBE, and 100 µl milliQ H_2_O. Lipid extracts were dried in a miVac centrifugal evaporator (Genevac Ltd., UK) and redissolved in 100 µl of water:acetonitrile (38:62).

The analyses of fatty acids in the samples were performed in a Dionex Ultimate 3000 HPLC system (Thermo Scientific, Bremen, Germany) equipped with a column Zorbax Eclipse Plus C18 (50 mm × 2.1 mm, 1.8 μm). Mobile phases A and B were water and acetonitrile, respectively, both containing 0.1% of formic acid. Fatty acids were separated in an injection volume of 2 µl by a gradient program as follows: 62% B (held for 4.5 min) followed by a linear increase up to 100% B in 10 min (held for 1 min). The column temperature was set at 45°C. Mass detection was carried out in a Bruker Impact II q-ToF mass spectrometer with electrospray ionization, operating in the negative mode. The settings of the mass spectrometer were as follows: spray voltage 4.5 kV; drying gas flow 12 l/min; drying gas temperature 250°C; and nebulizer pressure 44 psi.

For quantification, calibration curves for each compound were prepared by dissolution of the pure standards in methanol to adequately encompass the expected concentration of the analytes in the samples. The calibration ranges were as follows: 0.4–12.5 µg/ml for EPA and γ-linolenic; 1.2–37.5 µg/ml for DHA and linolenic; 2.3–75 µg/ml for AA and palmitoleic; 3.9–125 µg/ml for linoleic; and 7.8–250 µg/ml for oleic, palmitic, and stearic acids. A good linearity was observed in all cases (*r*^2^ > 0.994). Heptadecanoic acid was used as internal standard to account for potential biases during the extraction protocol.

### Analysis of Fecal Microbiota

Fecal sample collection and metagenomic analyses of fecal microbiota were performed as previously reported (22). Briefly, fresh fecal material was processed within 3 h from collection and immediately homogenized and stored at −80°C. Fecal DNA was extracted with a QIAampDNA stool minikit (Qiagen, Strasse, Germany). Then, 16S rRNA gene sequences were amplified, and 16S rRNA and gene-based amplicons were sequenced by an Ion Torrent PGM sequencing platform as described elsewhere (22).

### Analysis of Short-Chain Fatty Acids (SCFA) in Fecal Samples

Analysis of SCFA (acetate, propionate, and butyrate) was performed by gas chromatography. Briefly, 1 g of fecal samples was diluted 1:10 in sterile PBS and homogenized in a LabBlender 400 stomacher (Seward Medical, London, UK) at full speed for 4 min. Then, supernatants were obtained by centrifugation (10.000 × *g*, 30 min, 4°C), filtered through 0.2-µm filters, mixed with 1:10 of ethyl butyric acid (2 mg/ml) as an internal standard, and stored at −80°C until analysis.

A gas chromatograph 6890N (Agilent Technologies Inc., Palo Alto, CA, USA) connected to a mass spectrometry (MS) 5973N detector (Agilent Technologies) and to a flame ionization detector was used for identification and quantification of SCFA. Data were collected using the Enhanced ChemStation G1701DA software (Agilent). Samples (1 µl) were injected into the gas chromatograph equipped with an HP-Innowax capillary column (60-m length by 0.25-mm internal diameter, with a 0.25-µm film thickness; Agilent) using He as a gas carrier (flow rate of 1.5 ml/min). The temperature of the injector was kept at 220°C, and the split ratio was 50:1. Chromatographic conditions were as follows: initial oven temperature of 120°C, 5°C/min up to 180°C, 1 min at 180°C, and a ramp of 20°C/min up to 220°C to clean the column. In the MS detector, the electron impact energy was set at 70 eV. The data collected were in the range of 25 to 250 atomic mass units (at 3.25 scans/s).

SCFA were identified by comparison of their mass spectra with those held in the HP-Wiley 138 library (Agilent) and by comparison of their retention times with those of the corresponding standards (Sigma Aldrich, St. Louis, MO, USA). The peaks were quantified as relative abundances with respect to the internal standard. The concentration (in millimolar) of each SCFA was calculated using the linear regression equations (*R*^2^ ≥ 0.99) from the corresponding standard curves.

### Analysis of Serum Biomarkers

#### Soluble Biomarkers

Serum levels of vascular endothelial growth factor (VEGF), granulocyte monocyte colony-stimulating factor (GM-CSF), and IL-8 were analyzed by Cytometric Bead Arrays (BD Biosciences, NJ, USA) using a BD FACS Canto II and FACS Diva software. Detection limits were 4.5, 0.2, and 1.2 pg/ml, respectively.

Epidermal growth factor (EGF), TNFα, MCP-1, interferon gamma-inducible protein-10 (IP-10), and leptin serum levels were assessed by plate immunoassays using commercial kits by Peprotech (NJ, USA), following manufacturer’s instructions. Detection limits were 3.9, 3.9, 8, 3.9, and 24 pg/ml, respectively.

#### Malondialdehyde (MDA)

Malondialdehyde serum levels were determined by means of a colorimetric method using a commercial kit (LPO-596, Byoxytech, Oxis International, France). Final absorbance was read at 586 nm.

### Anthropometric Measures

Height was measured using a stadiometer with an accuracy of ±1 mm (Año-Sayol, Barcelona, Spain). The subjects stood barefoot, in an upright position and with the head positioned in the Frankfort horizontal plane. Weight was measured on a scale with an accuracy of ±100 g (Seca, Hamburg, Germany).

### Nutritional Assessments

Dietary intakes were assessed by means of an annual semiquantitative validated food frequency questionnaire including 160 items (25). During an interview by trained dietitians, subjects were asked, item by item, whether they usually ate each food and, if so, how much they usually ate. For this purpose, three different serving sizes of each cooked food were presented in pictures to the participants so that they could choose from up to seven serving sizes (from “less than the small one” to “more than the large one”). For some of the foods consumed, amounts were recorded in household units, by volume, or by measuring with a ruler. Information on the cooking practices, number and amount of ingredients used in each recipe, and other relevant information for the study was collected. Methodological issues concerning dietary assessment have been detailed elsewhere (25). The consumption of foods was converted into energy intake (kilocalories per day), macronutrients (carbohydrates, lipids, and proteins, grams per day), and total fiber (grams per day) using the nutrient food composition tables developed by the Centro de Enseñanza Superior de Nutrición Humana y Dietética (CESNID) (26). CESNID, a foundation, involves different institutions, universities, and companies related to the food and nutrition area, and its food composition databases are supported by the Spanish Association of Nutrition and Dietetics.

### Statistical Analyses

Continuous variables were expressed as median (interquartile range) or mean ± SD. Mann–Whitney *U*, Student’s *t* or Kruskal–Wallis tests were performed to assess statistical differences. Correlations were analyzed by Spearman’s rank or Pearson tests, depending on the distribution of the data. Categorical variables were summarized as *n* (%), and differences were analyzed by χ^2^ tests. A principal component analysis (PCA) was performed to avoid potential bias due to collinearity. The adequacy of the data was studied by Kaiser–Meyer–Olkin test and Bartlett test of sphericity. The number of components retained was based on eigenvalues (>1), and loadings greater than 0.5 were used to identify the variables comprising each component. Unsupervised cluster analysis was performed based on squared Euclidean distances, and Ward’s Minimum Variance method was used to identify the clusters. Heatmaps were generated under R package *heatmap.2*. SPSS 21.0, R 3.0.3, and GraphPad Prism 5.0 for Windows were used.

## Results

### Total FFA Serum Levels in SLE Patients: Association with Intestinal Dysbiosis

The concentration of total FFA was measured in serum samples from 21 SLE patients and 25 matched HC (Table 1). SLE patients exhibited higher FFA serum levels (Table 1). Differences between groups in the levels of FFA remained significant after adjusting for age and gender (*p* = 0.024). Moreover, no associations were found with demographical parameters, cholesterol and triglycerides levels, and dietary intakes (Table 2). Furthermore, FFA were neither related to clinical manifestations (Table 3) (all *p* > 0.050) nor disease activity (*r* = −0.349, *p* = 0.169), duration (*r* = −0.005, *p* = 0.982), or anti-dsDNA levels (*r* = −0.350, *p* = 0.130) in SLE. Therefore, parameters other than those indicated may explain the altered serum FFA levels registered in SLE.

**Table 1:** **Serum-free fatty acids (FFA) levels, nutritional parameters and blood lipid profiles of the healthy controls (HC) and systemic lupus erythematosus (SLE) patients recruited in this study**.

	HC (*n* = 25)	SLE (*n* = 21)	*p*
**FFA assessment**			
Total FFA (mM)	0.27 (0.17)	0.41 (0.26)	0.045
**Blood lipid analyses**			
Total cholesterol (mg/dl)	191.50 (49.00)	200.00 (61.25)	0.732
HDL-cholesterol (mg/dl)	62.00 (14.75)	62.00 (24.00)	0.740
LDL-cholesterol (mg/dl)	114.50 (50.00)	111.50 (57.50)	0.530
Triglycerides (mg/dl)	68.50 (54.25)	71.55 (45.25)	0.715
**Nutritional parameters**			
Total energy (kcal/day)	1,888.88 (226.53)	2,186.11 (208.16)	0.069
Carbohydrates (g/day)	202.50 (51.60)	205.46 (102.95)	0.944
Lipids (g/day)	78.13 (29.32)	79.42 (63.57)	0.789
Proteins (g/day)	96.98 (15.81)	102.60 (34.30)	0.782
Fiber (g/day)	24.68 (6.17)	26.47 (6.57)	0.609
Body mass index (kg/m^2^)	24.96 (4.47)	24.58 (7.78)	0.715

*Variables are represented as median (interquartile range) or *n* (%) unless otherwise stated. Differences in demographical and blood lipid variables were assessed by Mann–Withney *U* tests, whereas differences in daily intakes were analyzed by multivariate analyses adjusted for confounders. Energy was adjusted by gender and age, whereas the rest of the nutrients were adjusted by gender, age, and energy*.

**Table 2:** **Analysis of the correlation between serum FFA levels and demographical and nutritional features in healthy controls (HC) and systemic lupus erythematous (SLE) patients**.

	HC	SLE
Age	*r* = −0.260	*r* = −0.179
	*p* = 0.231	*p* = 0.451
Total cholesterol	*r* = −0.108	*r* = −0.203
	*p* = 0.625	*p* = 0.390
HDL-cholesterol	*r* = 0.220	*r* = 0.114
	*p* = 0.313	*p* = 0.633
LDL-cholesterol	*r* = −0.028	*r* = −0.248
	*p* = 0.898	*p* = 0.291
Triglycerides	*r* = −0.224	*r* = −0.078
	*p* = 0.305	*p* = 0.743
BMI	*r* = 0.247	*r* = 0.214
	*p* = 0.268	*p* = 0.366
Total energy	*r* = 0.082	*r* = −0.212
	*p* = 0.710	*p* = 0.369
Carbohydrates	*r* = −0.107	*r* = −0.311
	*p* = 0.628	*p* = 0.182
Lipids	*r* = −0.048	*r* = −0.220
	*p* = 0.826	*p* = 0.352
Proteins	*r* = 0.010	*r* = −0.005
	*p* = 0.964	*p* = 0.985
Fiber	*r* = 0.125	*r* = −0.394
	*p* = 0.568	*p* = 0.086

*Correlations were assessed by Spearman ranks tests (*r* coefficient and *p*-value is indicated for each parameter)*.

**Table 3:** **Demographical and clinical parameters of the systemic lupus erythematosus (SLE) patients**.

	SLE (*n* = 21)
Age, (years), mean (range)	48.35 (27.00–70.00)
Gender, (f/m)	21/0
Age at diagnosis, (years)	33.00 (14.50)
Disease duration, (years), median (range)	7.00 (2.00–24.00)
SLEDAI score	4.00 (3.25)
**Clinical manifestations, *n*(%)**	
Malar rash	12 (57.1)
Photosensitivity	16 (76.2)
Discoid lesions	6 (28.6)
Arthritis	10 (47.6)
Oral ulcers	10 (47.6)
Serositis	4 (19.0)
Renal disorder	3 (14.3)
Neurological disorder	0 (0.0)
Cytopenia	11 (52.4)
**Autoantibodies, *n*(%)**	
ANAs	21 (100)
Anti-dsDNA titer, (U/ml), mean ± SD	25.30 ± 33.89
Anti-SSa	11 (52.4)
Anti-SSb	2 (9.5)
Anti-Sm	2 (9.5)
Anti-RNP	1 (4.8)
**Treatments, *n* (%)**	
None or NSAIDs	3 (14.2)
Antimalarials	18 (85.7)

*Variables are represented as median (interquartile range) or *n*(%), unless otherwise stated*.

**Figure 1:**
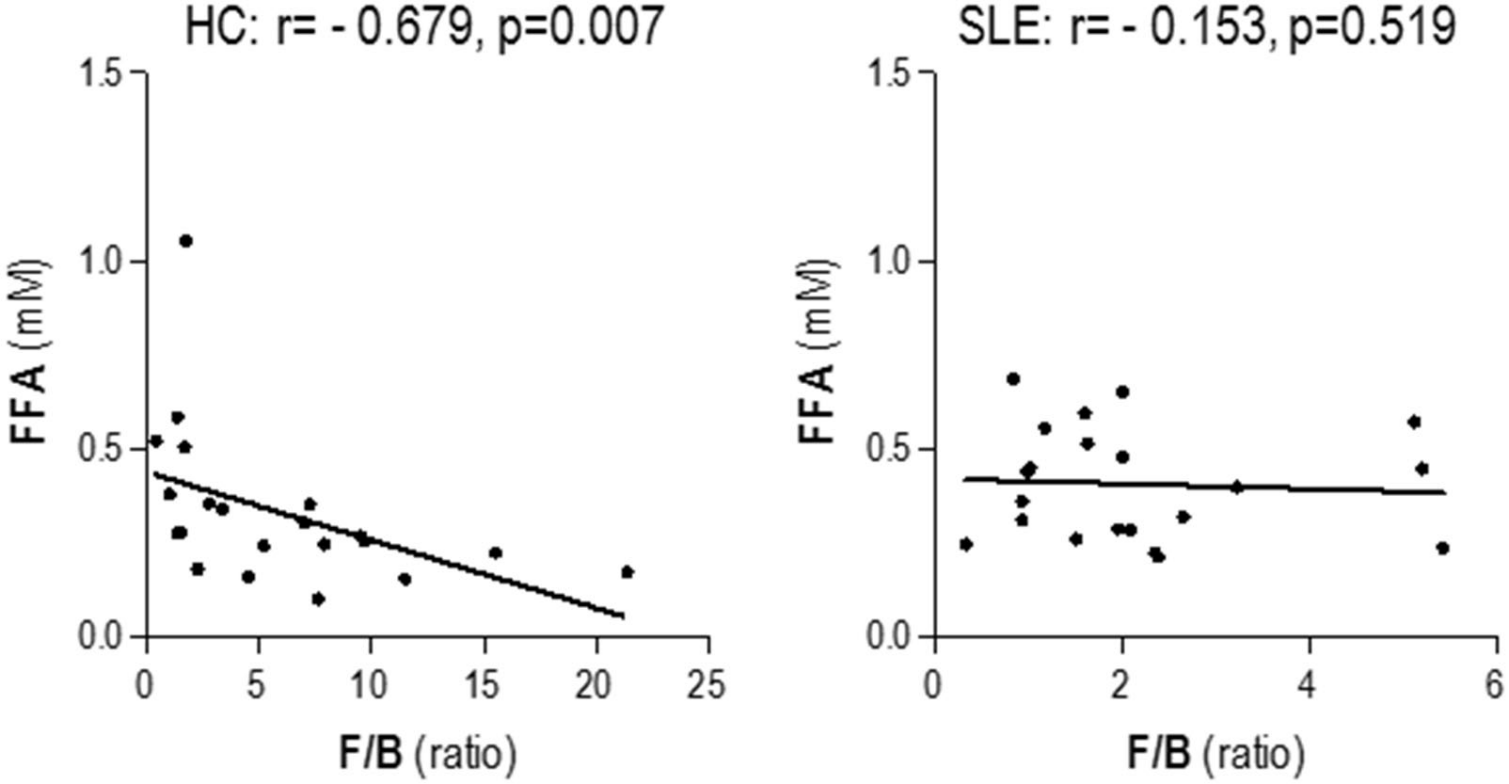
**Serum-free fatty acids (FFA) levels and *Firmicutes* to *Bacteroidetes* (F/B) ratio**. The association between serum FFA levels and the F/B ratio was analyzed by Spearman’s ranks correlation tests.

Then, we wondered whether gut microbial composition may account for the increased FFA serum levels in SLE. To this aim, the associations between FFA levels and the main intestinal microbial groups analyzed by a metagenomic approach as already described (22) were assessed. As previously reported, diminished *Firmicutes* to *Bacteroidetes* (F/B) ratio characterized the intestinal dysbiosis found in SLE compared to healthy subjects [1.94 (1.51) vs 4.27 (5.93), *p* < 0.001] (22). Among the phyla analyzed (*Actinobacteria, Bacteroidetes, Firmicutes, Cyanobacteria, Euryarchaeota, Fusobacteria, Lentisphaerae, Proteobacteria, Tenericutes*, TM7, *Verrucomicrobia*, and *Synergistetes*), FFA levels displayed opposite correlations with the *Firmicutes* and *Bacteroidetes* groups in HC, but not in lupus patients (Table 4). Consequently, a negative association with the F/B ratio was observed in HC, but not in SLE (Figure 1). Moreover, this association remained significant after adjusting for potential confounders (Table 5). Therefore, F/B ratio was found to be the main predictor of FFA serum levels in HC, whereas this effect was not seen in lupus patients, hallmarked by a decreased F/B ratio and elevated FFA levels.

**Table 4:** **Association between serum-free fatty acids (FFA) levels and gut microbiota composition in healthy controls (HC) and systemic lupus erythematosus patients (SLE)**.

	HC	SLE)
*Actinobacteria*	*r* = 0.075	*r* = −0.415
	*p* = 0.733	*p* = 0.069
*Bacteroidetes*	***r* = 0.721**	*r* = 0.311
	***p* < 0.0001**	*p* = 0.182
*Firmicutes*	***r* = −0.574**	*r* = −0.117
	***p* = 0.007**	*p* = 0.622
*Cyanobacteria*	*r* = −0.120	*r* = −0.401
	*p* = 0.539	*p* = 0.080
*Euryarchaeota*	*r* = −0.213	*r* = −0.063
	*p* = 0.328	*p* = −0.792
*Fusobacteria*	*r* = −0.052	*r* = 0.139
	*p* = 0.812	*p* = 0.560
*Lentisphaerae*	*r* = 0.272	*r* = 0.019
	*p* = 0.210	*p* = 0.937
*Proteobacteria*	*r* = −0.158	*r* = 0.230
	*p* = 0.471	*p* = 0.329
*Tenericutes*	*r* = −0.105	*r* = −0.220
	*p* = 0.634	*p* = 0.227
TM7	*r* = 0.015	*r* = 0.018
	*p* = 0.945	*p* = 0.939
*Verrucomicrobia*	*r* = −0.325	*r* = 0.209
	*p* = 0.130	*p* = 0.376
*Synergistetes*	*r* = −0.211	*r* = −0.104
	*p* = 0.334	*p* = 0.661

*The associations between serum FFA levels and the abundance of microbial groups at the level of phyla in HC and SLE patients were analyzed by Spearman ranks tests (*r* coefficient and *p*-value is indicated for each parameter). Statistical analyses with a *p*-value below 0.050 are highlighted in bold*.

**Table 5:** ***Firmicutes/Bacteroidetes* (F/B) ratio is the main predictor of FFA levels in healthy controls (HC) but not in systemic lupus erythematosus (SLE) patients**.

		β	*B* [95% CI]	*p*
HC	F/B ratio	−0.636	−0.334 [−0.557, −0.111]	**0.007**
	Age	−0.354	−0.652 [−1.436, 0.132]	0.093
	Gender	0.150	0.114 [−0.179, 0.407]	0.405
	BMI	−0.039	−0.002 [−0.022, 0.018]	0.832
	CRP	0.191	0.133 [−0.170, 0.436]	0.352
	Total energy	−0.010	0.001 [−0.044, 0.042]	0.961
	Lipids	0.276	0.003 [−0.004, 0.010]	0.315
	Carbohydrates	−0.372	−0.002 [−0.004, 0.001]	0.073
	Proteins	0.092	0.001 [−0.005, 0.007]	0.690
	Fiber	−0.223	−0.007 [−0.020, 0.005]	0.239
SLE	F/B ratio	−0.025	−0.014 [−0.332, 0.303]	0.923
	Age	−0.229	−0.336 [−1.315, 0.643]	0.466
	BMI	0.415	0.012 [−0.008, 0.032]	0.205
	CRP	0.186	0.072 [−0.191, 0.335]	0.558
	Total energy	−0.072	−0.007 [−0.045, 0.031]	0.687
	Lipids	−0.193	−0.001 [−0.005, 0.003]	0.698
	Carbohydrates	−0.265	−0.001 [−0.003, 0.002]	0.649
	Proteins	0.369	0.002 [−0.002, 0.006]	0.308
	Fiber	−0.351	−0.006 [−0.019, 0.008]	0.365

*The association between F/B ratio and FFA serum levels in HC was studied by multiple lineal regression analysis including demographical parameters and nutritional intakes as potential confounders. HC: *R*^2^ (model) = 0.762; SLE: *R*^2^ (model) = 0.426. Statistical analyses with a *p*-value below 0.050 are highlighted in bold*.

**Figure 2:**
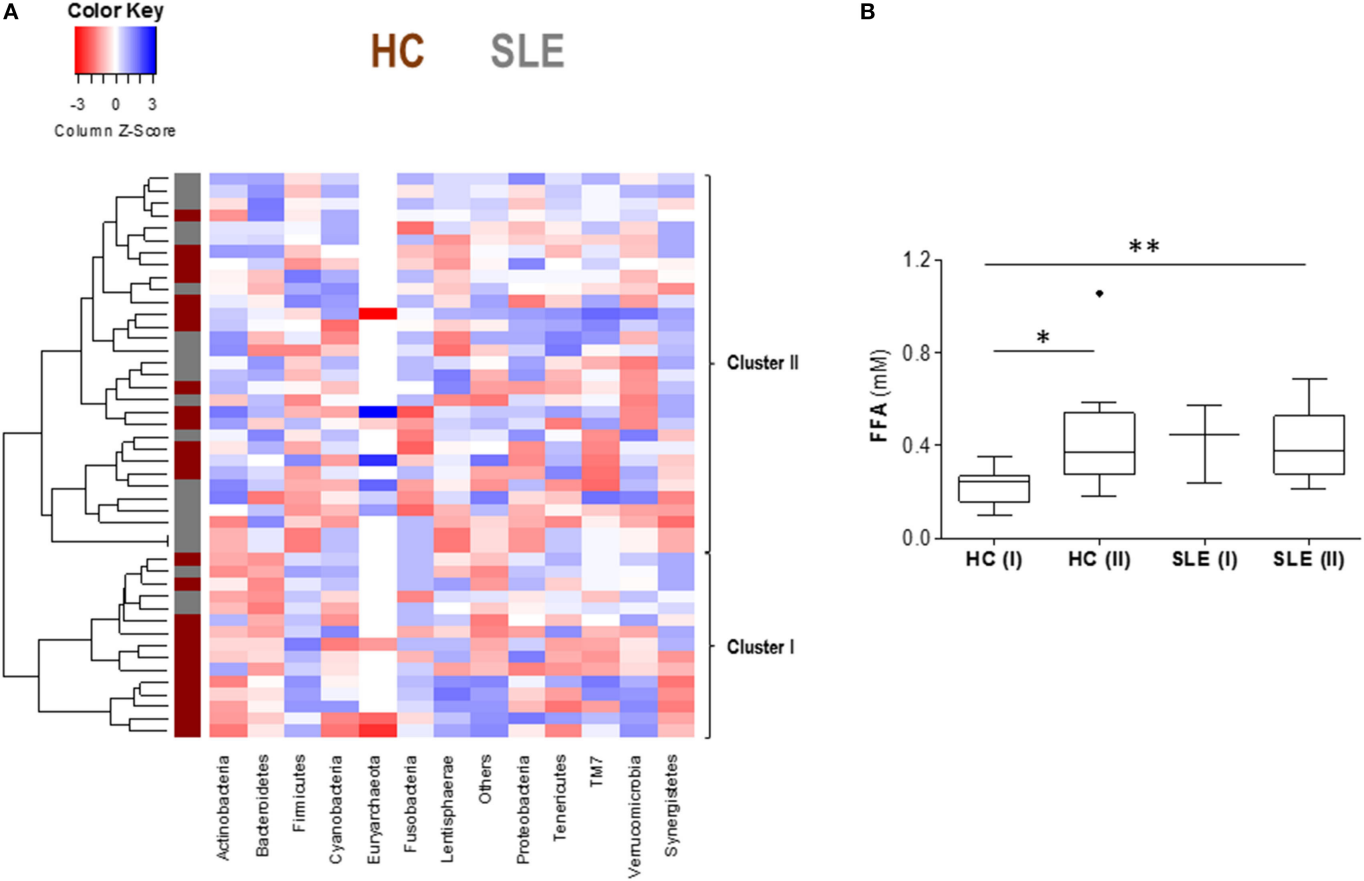
**Cluster analysis revealed a link between gut microbiota composition and total free fatty acids (FFA) serum levels**. **(A)** Heatmap plotting gut microbiota composition, based on the main phyla, in healthy controls (HC) and systemic lupus erythematosus (SLE) patients. Tiles are colored based on the abundance of each group, red and blue colors indicating low or high levels, respectively. Each row represents an individual, and the vertical colored bar at the left represents HC (dark red) or SLE patient (gray). Two clusters were identified and are highlighted at the right (clusters I and II). **(B)** Comparison of total FFA serum levels among groups after stratifying by disease status (HC or SLE) and microbiota clusters (I or II). Boxes represent median and interquartile range, whereas whiskers represent minimum and maximum values. Differences were assessed by Kruskal–Wallis test and Dunn–Bonferroni *post hoc* correction for multiple comparisons tests. *p < 0.050, **p < 0.010.

Since some heterogeneity within groups in the FFA levels was observed and in order to gain more insight into the connections between gut microbiota and serum FFA, further analyses were performed. Focusing on the main microbial groups at the level of phyla, individuals were classified into groups by means of a cluster analysis. Interestingly, two main clusters were identified (thereafter referred to as clusters I and II) (Figure 2A), mainly differing in the F/B ratio (8.84 ± 5.53 vs 1.70 ± 0.76, respectively; *p* < 0.0001). Notably, a distinct distribution of individuals was observed, as HC were mainly found within the cluster I (14/25), whereas SLE patients were marginally present in this group (3/21, *p* = 0.004). On the one hand, this result highlights a shift in the microbiota composition in SLE patients compared to HC, hence supporting an association between a biased distribution of the intestinal microbial groups and elevated FFA serum levels. More importantly, when FFA levels were compared among HC and SLE subjects stratified by microbial clusters, it was noted that HC grouping within the cluster II exhibited similar FFA serum levels as SLE patients (Figure 2B), thus reinforcing the relevance of the microbiota composition on the FFA serum levels.

Overall, our findings disclose a strong association between FFA levels in serum and specific groups of the gut microbiota in healthy individuals, but not in lupus patients where a profound intestinal dysbiosis was registered.

### SCFA and FFA Levels

Our results point to a relationship between the gut microbiota and the host metabolism at the systemic level, but the actual mediators are unclear. Since SCFA may affect the human metabolism and an altered gut microbiota composition leads to a dysregulated SCFA production, the associations between fecal SCFA levels and those of serum FFA were analyzed.

On the one hand, higher levels of all SCFA studied were observed in lupus patients compared to HC (Table 6). However, no differences were found between SLE and HC when relative proportions were compared (all *p* > 0.050). On the other hand, all SCFA exhibited a positive correlation with FFA serum levels in HC (Table 6).

**Table 6:** **Analysis of fecal short-chain fatty acids (SCFA) levels and their correlation with serum-free fatty acids (FFA) levels in healthy controls (HC) and systemic lupus erythematosus (SLE) patients**.

	HC	SLE	*p*
**SCFA levels (mM)**			
Acetate	41.14 (12.30)	57.63 (19.63)	**0.005**
Propionate	11.96 (8.72)	20.61 (9.80)	**0.003**
Butyrate	7.78 (3.97)	10.50 (7.14)	0.075
**SCFA–FFA correlations**			
Acetate	***r* = 0.770**	*r* = 0.067	
	***p* < 0.001**	*p* = 0.793	
Propionate	***r* = 0.790**	*r* = 0.230	
	***p* < 0.001**	*p* = 0.359	
Butyrate	***r* = 0.764**	*r* = −0.066	
	***p* < 0.001**	*p* = 0.795	

*The differences between fecal SCFA levels found in lupus patients and those in HC were analyzed by Mann–Withney *U* tests; whereas the correlation analyses were performed by Spearman rank’s tests. Variables are summarized as median (interquartile range). Statistical analyses with a *p*-value below 0.050 are highlighted in bold*.

**Figure 3:**
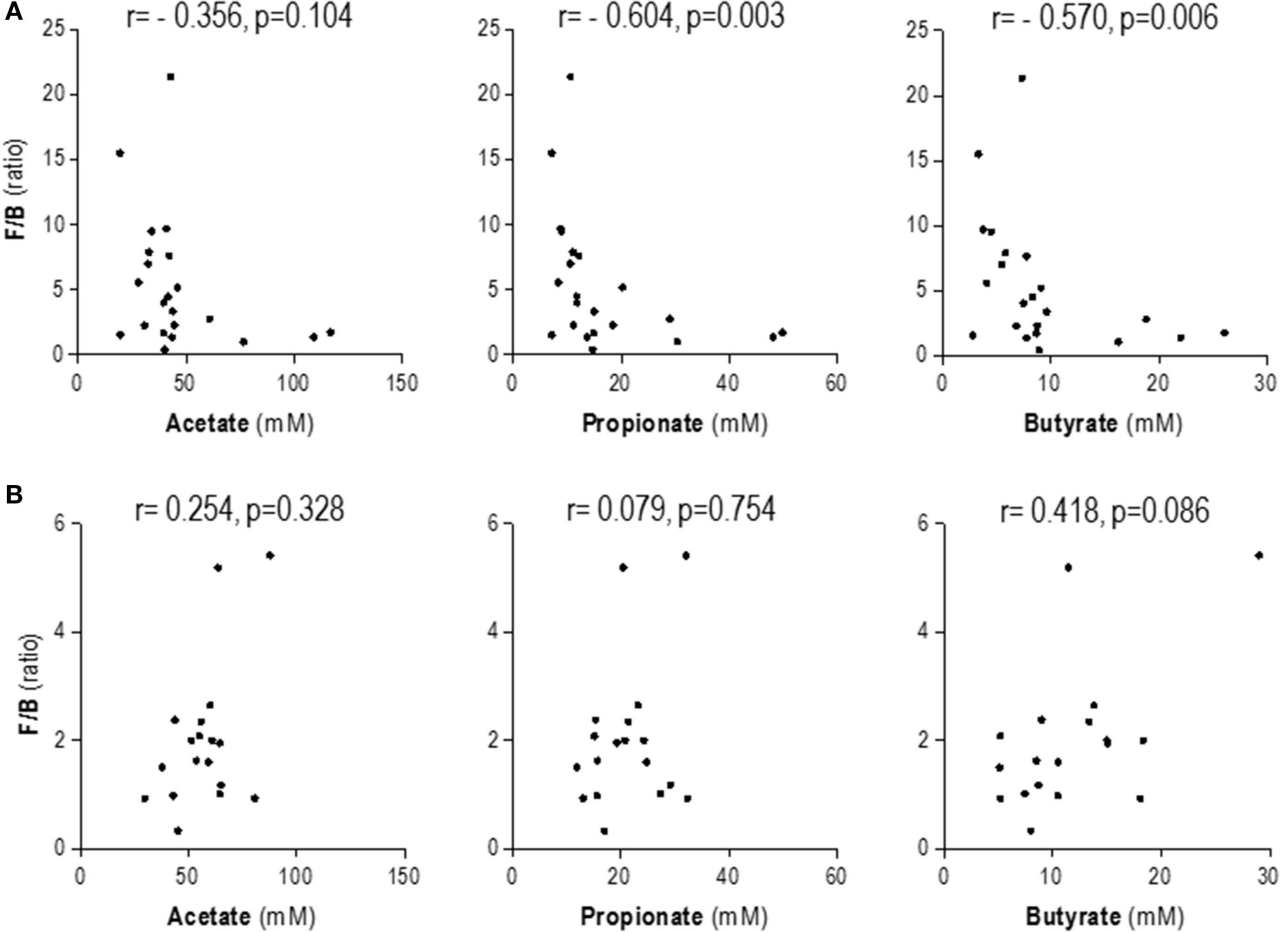
**Association between fecal short-chain fatty acids (SCFA) levels and *Firmicutes* to *Bacteroidetes* ratio**. Correlation analyses by Spearman’s ranks tests were performed in order to analyze the association between fecal SCFA levels and those of serum-free fatty acids in healthy controls **(A)** and systemic lupus erythematosus patients **(B)**.

Importantly, the F/B ratio was negatively correlated to the fecal levels of propionate and butyrate in HC but not in SLE patients (Figure 3). Notably, stronger associations were found in HC when only *Bacteroidetes* abundance was considered: propionate (*r* = 0.653, *p* < 0.001) and butyrate (*r* = 0.623, *p* = 0.002).

In order to gain further insight into the relevance of the intestinal microbiota and the SCFA production and serum FFA levels, we performed additional analyses by stratifying subjects according to the clusters obtained from the microbiota analysis (Figure 2) instead of their clinical condition. Interestingly, a negative association between F/B ratio and fecal SCFA was found in cluster I (propionate: *r* = −0.621, *p* = 0.024 and butyrate: *r* = −0.654, *p* = 0.015), but not in those grouped in cluster II (propionate: *r* = −0.015, *p* = 0.940 and butyrate: *r* = 0.220, *p* = 0.271). Overall, this picture mirrors that of found for the HC vs SLE populations according to our previous findings and confirms a pivotal role of the intestinal microbiota in this scenario. However, SCFA and FFA levels did not remain associated after stratifying the whole population by the clusters, hence suggesting the involvement of additional factors, such as the clinical condition, to explain the connection between gut microbiota composition, SCFA, and serum FFA levels.

All these results highlight a role for the gut microbiota in the maintenance of serum FFA levels, SCFA having a potential role orchestrating this interaction. Additional factors, such as disease status, may also influence the outcome of the associations between gut microbiota composition and the interaction SCFA–FFA. Indeed, altered gut microbiota composition found in SLE patients was linked to an altered SCFA production and increased FFA levels in serum, thus supporting this hypothesis.

### FFA Profiling in SLE Patients

Although elevated FFA levels were found in SLE, whether a global increase in all FFA species underlies this finding, or if some specific FFA were altered was not clear. To address this issue, a number of FFA species were measured, and the differences between SLE and HC were studied.

**Figure 4:**
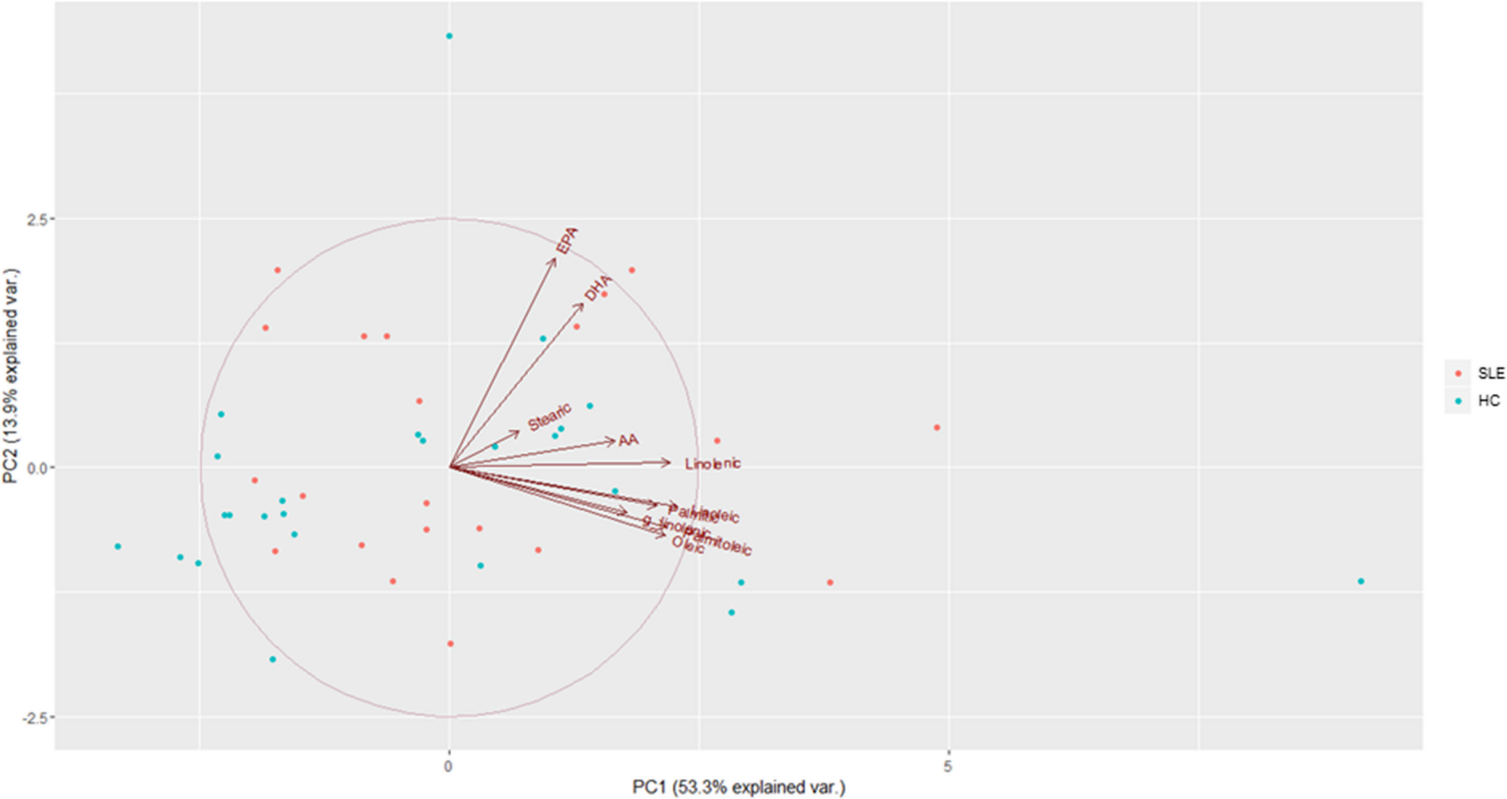
**Principal component analysis (PCA) of specific free fatty acids (FFA) profiling**. Biplot obtained in the PCA of specific FFA serum levels. PC1 and PC2 are represented in the horizontal and vertical axes, respectively. Arrows represent the vectors showing the associations among the raw variables entered in the analysis. Individuals are represented in colored dots: healthy controls (HC) (turquoise) and systemic lupus erythematosus (SLE) (red).

Overall, no striking differences were observed between patients and controls (Table 7). However, since some collinearity among FFA species existed, a PCA was carried out on the FFA species analyzed to avoid potential biases. PCA demonstrated a good adequacy of the data (KMO statistic = 0.781, Barlett sphericity test *p* = 10^−44^), and three components were identified (eigenvalues >1) explaining 77.53% of the total variance. Based on their loadings, PC1 (53.32% variance explained) retained γ-linolenic, palmitoleic, palmitic, oleic, linolenic, linoleic, and arachidonic acids, and PC2 (13.95% variance explained) mainly retained EPA and DHA. Finally, PC3 (10.26% variance explained) only retained stearic acid (Figure 4).

**Table 7:** **Specific free fatty acids (FFA) in healthy controls (HC) and systemic lupus erythematosus (SLE) patients**.

FFA (μg/ml)	HC	SLE	*p*
Palmitic (16:0)	32.76 (15.59)	30.35 (8.37)	0.982
Stearic (18:0)	28.51 (12.95)	29.12 (6.19)	0.873
Palmitoleic (16:1ω7)	1.93 (1.62)	2.67 (0.99)	0.351
Oleic (18:1ω9)	27.76 (20.61)	34.39 (17.77)	0.467
Linoleic (18:2ω6)	6.99 (6.47)	10.26 (7.35)	0.246
γ-Linoleic (18:3ω6)	0.08 (0.08)	0.10 (0.06)	0.785
AA (20:4ω6)	1.96 (1.68)	2.74 (1.74)	**0.045**
Linolenic (18:3ω3)	0.18 (0.17)	0.20 (0.16)	0.539
EPA (20:5ω3)	0.07 (0.15)	0.15 (0.17)	0.363
DHA (22:6ω3)	1.47 (1.46)	1.65 (1.76)	0.209

*The differences in specific FFA serum levels between control and patients were assessed by Mann–Withney *U* tests. Variables are summarized as median (interquartile range). Statistical analyses with a *p*-value below 0.050 are highlighted in bold*.

On the one hand, these results underlie the outstanding heterogeneity of FFA species, a clearly distinct pattern of grouping depending on their chemical structure (chain length or double-bond position) not being found. Globally, saturated, monounsaturated, and w6 fatty acids were grouped together within the PC1, whereas the most important anti-inflammatory w3 FFA did in the PC2. Stearic acid, of controversial immunological and metabolic role, was grouped in the third component. Hence, these findings support a functional, rather than structural, relationship of FFA.

On the other hand, no differences in the PCA scores were registered between SLE and HC groups (PC1: *p* = 0.169, PC2: *p* = 0.378, and PC3: *p* = 0.916), thus suggesting that SLE patients did not exhibit a different FFA profile compared to HC.

Finally, whether PCA scores could be related to gut microbiota composition was analyzed. Notably, F/B ratio was positively correlated with the PC2 score (Table 8) in HC but not in SLE, thereby suggesting a beneficial effect of gut microbiota composition on the serum FFA pool in healthy individuals. Again, when the phyla were independently studied, *Bacteroidetes* exhibited a stronger correlation with PC2 score (*r* = −0.433, *p* = 0.039) than that of *Firmicutes* (*r* = 0.411, *p* = 0.052) in HC individuals. Thus, gut microbiota seems to quantitatively and qualitatively impact the FFA serum pool.

**Table 8:** **Associations between *Firmicutes* to *Bacteroidetes* ratio and free fatty acids-principal component analysis scores in healthy controls (HC) and systemic lupus erythematosus (SLE) patients**.

	HC	SLE
PC1	*r* = −0.246	*r* = 0.078
	*p* = 0.257	*p* = 0.736
PC2	***r* = 0.437**	*r* = 0.110
	***p* = 0.037**	*p* = 0.635
PC3	*r* = −0.075	*r* = 0.089
	*p* = 0.734	*p* = 0.700

*Correlations between these parameters were assessed by Pearson correlation tests. Statistical analyses with a p-value below 0.050 are highlighted in bold*.

### FFA and Serum Biomarkers in SLE Patients

Since some associations between gut microbiota and specific FFA PCA scores were observed, we aimed to evaluate whether these parameters could be related to some relevant serum biomarkers in lupus. To this end, a panel of serum biomarkers of endothelial activation (VEGF, GM-CSF, EGF, IL-8, TNFα, MCP-1, IP-10, and leptin) and oxidative stress (MDA) were measured (Table 9).

**Table 9:** **Levels of serum biomarkers analyzed in healthy controls (HC) and systemic lupus erythematosus (SLE) patients**.

	HC	SLE	*p*
Vascular endothelial growthfactor [VEGF] (pg/ml)	83.38 (49.49)	70.73 (98.87)	0.326
Granulocyte monocyte colonystimulating factor [GM-CSF] (pg/ml)	0.35 (0.92)	0.35 (2.77)	0.424
Epidermal growth factor [EGF] (pg/ml)	114.29 (81.35)	65.57 (78.61)	**0.019**
Interleukin-8 [IL-8] (pg/ml)	14.69 (22.51)	27.85 (21.92)	**0.016**
Tumor necrosis factor alpha [TNFα] (pg/ml)	174.40 (309.4)	188.14 (292.92)	0.789
Monocyte chemoattractantprotein-1 [MCP-1] (pg/ml)	444.55 (481.45)	616.13 (402.07)	**0.011**
Interferon gamma-inducibleprotein-10 [IP-10] (pg/ml)	98.82 (148.81)	167.67 (152.76)	**0.019**
Leptin (ng/ml)	7.73 (7.14)	14.16 (20.20)	**<0.001**
Malondialdehyde [MDA] (μM)	2.78 (0.71)	2.90 (0.44)	0.658

*The differences in serum levels between control and patients were assessed by Mann–Withney *U* tests. Variables are summarized as median (interquartile range). Statistical analyses with a *p*-value below 0.050 are highlighted in bold*.

First, the associations between these biomarkers and the gut microbiota composition were analyzed. Interestingly, the F/B ratio was negatively associated to leptin (*r* = −0.369, *p* = 0.006) and MCP-1 serum levels (*r* = −0.304, *p* = 0.025) in the whole group.

On the other hand, divergent associations were noted among FFA PCA scores and these biomarkers in SLE patients. Whereas PC1 was positively correlated with biomarkers of endothelial activation [VEGF (*r* = 0.444, *p* = 0.044), IL-8 (*r* = 0.522, 0.015), and EGF (*r* = 0.400, *p* = 0.070)], negative associations were observed for PC2 [EGF (*r* = −0.425, *p* = 0.055), MCP-1 (*r* = −0.640, *p* = 0.002), IP-10 (*r* = −0.397, *p* = 0.075), TNFα (*r* = −0.410, *p* = 0.065), and MDA (*r* = −0.375, *p* = 0.057)]. Interestingly, negative associations were also found for PC3 [VEGF (*r* = −0.534, *p* = 0.013) and IL-8 (*r* = −0.459, *p* = 0.036)]. No associations were observed in the HC.

All these findings seem to point to a link between gut microbiota, FFA serum pool, and biomarkers of endothelial activation in lupus, thus emphasizing the systemic effect of the gut microbiota in this condition. Additionally, differences among FFA PCA are in line with their proposed functional diversity.

## Discussion

Over the last decade, several studies have revealed a number of interactions between the gut microbiota and the host in homeostatic conditions. Accordingly, dysbiosis has been consistently related to different pathological situations, from immune-mediated to metabolic diseases (27–29). In this sense, we have recently reported the existence of an intestinal dysbiosis in SLE (22). Additional studies from our group allowed us to associate the dysbiotic state with the dysregulated Treg/Th17 responses found in lupus patients (30). In the present report, we go a step further by addressing the study of the potential connections between the intestinal dysbiosis and the metabolic impairment in SLE, focusing on the role of FFA. Thus, gut microbiota may not only be related to disease pathogenesis itself but also to some comorbidities frequently present in lupus patients. Since the origin of such alterations is ill-defined, these new findings allow us to gain some insight into this complex situation and may help to delineate new therapeutic targets. Actually, the experimental modulation of the gut microbiota has yielded promising results in other scenarios (17). In fact, the lupus-like immune over-activation was partially reestablished *in vitro* by the supplementation with specific bacterial strains (30). Thus, our study warrants future research to assess whether this therapy may be also advisable to counteract the metabolic alterations in SLE patients.

Metabolic disorders, including metabolic syndrome, are common hallmarks of SLE and other related diseases (31–33). However, the underlying causes of these traits are not well defined. The findings herein presented suggest that gut microbiota may play a role in this condition. This notion can explain why a wide range of diseases, with striking differences in their clinical presentations, are associated with similar comorbidities (such as metabolic alterations). Thus, it is feasible to think that similar patterns of intestinal dysbiosis may underlie this situation. This idea is supported by the altered F/B ratios that were reported in other diseases exhibiting increased serum FFA levels (34–36). The fact that this ratio is a continuum may explain the differences in prevalence and severity of metabolic complications among different conditions. However, it must be remarked that studies on the alterations in the F/B ratio have yielded contradictory results in different contexts, such as obesity (37–39). Schwiertz and colleagues have recently published a reduced F/B ratio in obese and overweighed individuals in a large study population (40). Similarly, the enrichment of *Firmicutes* in the intestinal ecosystem has been related to improved lipid absorption and homeostasis in animal models (41). It must be taken into account that differences in sequencing techniques, data analysis, and characteristics of the recruited populations may be an important source of discrepancy in this field. Thus, the associations of the microbiota with metabolic traits in different scenarios must be interpreted with caution, since direct comparison is not always possible. Additional studies focusing on the F/B ratio in non-obese healthy population are warranted.

The association between the F/B ratio and the serum levels of FFA emphasizes the ability of the gut microbiota to promote systemic responses in the host. Moreover, it supports that gut microbiota can modulate the energy metabolism of the host (42). However, the identification of the actual mediators involved and the potential impact of this interaction in pathological conditions is currently lacking in the literature. Our findings in the present work point to the SCFA as potential orchestrators of the cross talk between gut microbiota and the host metabolism. Although these compounds are thought to be of key relevance in the interactions between intestinal microbial populations and the host (16), their link with the lipid metabolism remains controversial. Our results suggest that the SCFA production paralleled the FFA serum levels in healthy individuals. However, this association was absent in the pathological framework of lupus, where an increased SCFA production, together with elevated FFA serum levels, was noted. These results are in line with those from other metabolic conditions [reviewed in Ref. (43)]. Additionally, our analyses revealed that this aberrant SCFA production can be linked to an altered microbial gut composition, as a reduced F/B ratio was related to increased SCFA fecal levels. In this scenario, the potential involvement of the propionate deserves a special mention. Propionate is mainly produced by *Bacteroidetes* species (44), thereby supporting its negative association with the F/B ratio observed. It is important to note that increased propionate fecal levels have been reported in obese individuals exhibiting a decreased F/B ratio (40). Furthermore, exposure of human intestinal organoids to propionate led to an upregulation of genes belonging to lipolytic pathways (45). Moreover, experimental evidence from animal models and *ex vivo* experiments with human material have identified a mechanistic link between exposure to propionate and increased lipolysis mediated by the increased expression of the enzyme lipoprotein lipase (46–48). Moreover, a homeostatic role for propionate on glucose metabolism and regulation of energy intake has also been proposed. However, which is the actual role for propionate in human diseases required further elucidation. Taken together, our finding may provide a possible explanation for the elevated FFA serum levels in SLE, altered SCFA production, and overrepresentation of *Bacteroidetes* in the intestinal microbiota playing a pivotal role. The stronger associations of *Bacteroidetes* alone compared with those of the F/B ratio are in line with this point.

Another important result of our study was the association between FFA and some biomarkers of endothelial activation. Because of their nature, FFA are considered as common mediators between immunity, inflammation, and metabolism. Although some authors have previously proposed a role for the gut microbiota in the etiology of metabolic alterations and CVD (49), the actual players are far from being clear. Interestingly, experimental studies revealed a mechanistic link between propionate and leptin expression by human adipose and omental tissues (50), which is in line with our results. Nevertheless, the exact significance of this finding *in vivo* is not known. Taking into account the effects of FFA on inflammation, oxidative stress, and expression of adhesion molecules (51–53), our results may support a role for FFA as a link between the (altered) gut microbiota, host metabolism, and disease status. It is interesting to note that differences among FFA in their ability to promote endothelial activation *in vivo* or *in vitro* have been demonstrated (54–56), which is in line with the associations found in our study. Importantly, these biomarkers are considered as early preclinical indicators of CVD development in the long term (57–60). Taking this into account, these associations may point to a very early role of the altered gut microbiota in the etiology of these complications. This is reinforced by the negative association between the intestinal dysbiosis in lupus and the levels of the protective IgM antibodies against phosphorylcholine (30). These antibodies are known to enhance apoptotic-cell clearance and induce anti-inflammatory pathways, explaining its negative association with markers of subclinical atherosclerosis (61) and CVD development (62) in lupus.

Finally, our approach did not identify a different FFA serum profile in SLE patients in comparison to HC. Although a similar pattern of grouping of FFA species in the PCA was observed in rheumatoid arthritis patients by our group (24), differences among FFA species between patients and controls were not observed in the case of lupus. Interestingly, no differences in plasma FFA profiling were observed in a previous study with lupus patients (63), although slight alterations in the polyunsaturated fatty acids were reported in those with a previous history of CVD. Our results are, at least in part, in line with these findings, although differences in the experimental procedures between both studies are important. It is worthy to note that our group of patients was characterized by a low disease activity, even in the absence of glucocorticoid and immunosuppressive drugs. Thus, a larger study involving SLE patients with a higher degree of disease activity is warranted. However, despite no differences being found in the specific FFA levels, the results from the PCA emphasize the heterogeneity among FFA classes and suggest that FFA, despite being mostly unaltered, can develop different roles under different milieu. This hypothesis is in line with current evidence in this field (64, 65) and stresses the underestimated significance of FFA as key mediators for the human health.

Due to the lack of direct mechanistic data in our approach, these results pose the question on whether the microbiota composition is responsible of the altered FFA levels or if, alternatively, increased FFA levels may lead to changes in the gut ecology. Based on the literature currently available, several research studies seem to align with the former idea. The fact that no changes in the gut microbiota were related to disease duration (in spite of the wide range of disease duration studied in the present report) is also in line with this idea, probably suggesting that intestinal dysbiosis could be present at the preclinical stages of the disease. Similarly, experiments of fecal transplantation in obese and lean mice also support the causative role of the microbiota in shaping the host metabolism (66). However, due to the role of FFA on inflammation, it is tempting to speculate that these molecules can prompt a shift toward a systemic pro-inflammatory state, which can, in turn, disrupt the intestinal microbiota. Experimental studies with mice have revealed that diet-induced obesity is accompanied by changes in the gut microbiota and damaged intestinal barrier (67, 68). Interestingly, a diet with high ω6 content resulted in intestinal dysbiosis in mice, the inflammatory pathways playing a crucial role (69). Moreover, ω3 fatty acids seem to counteract the effects of diet- or antibiotics-induced dysbiosis through different mechanisms (70, 71). Additionally, an antibacterial effect was observed for some FFA (72). Therefore, it is feasible that a bidirectional cross talk between the gut microbiota and host metabolism is established, with immune circuits participating in this interaction.

In summary, our data indicate that increased serum FFA levels in SLE patients may be associated with changes in the gut ecosystem in the framework of lupus dysbiosis. The association between serum FFA and SCFA supports this notion. Additionally, different associations between FFA species and serum biomarkers of endothelial activation were found, hence not only underscoring the heterogeneity among FFA compounds but also shedding new light on the gut–metabolism–CVD axis. Although the reduced sample size and the lack of a mechanistic data are the limitations of our study, to the best of our knowledge this is the first report supporting a connection between gut microbiota, FFA, and biomarkers of endothelial activation. Moreover, we have provided a proof of concept evidence on the involvement of the intestinal dysbiosis in the metabolic alterations in lupus.

## Author Contributions

All the authors listed made substantial contributions to the design of the work, analysis, or interpretation of the results obtained; involved in drafting the manuscript, revising it critically for intellectual content, and approving the final version; and agreed to be accountable for all aspects of the work in ensuring that questions related to the accuracy or integrity of any part of the work are appropriately investigated and resolved.

## Conflict of Interest Statement

The authors declared no financial conflicts of interest. The funders have no role in study design, data analysis, or decision to publish.
